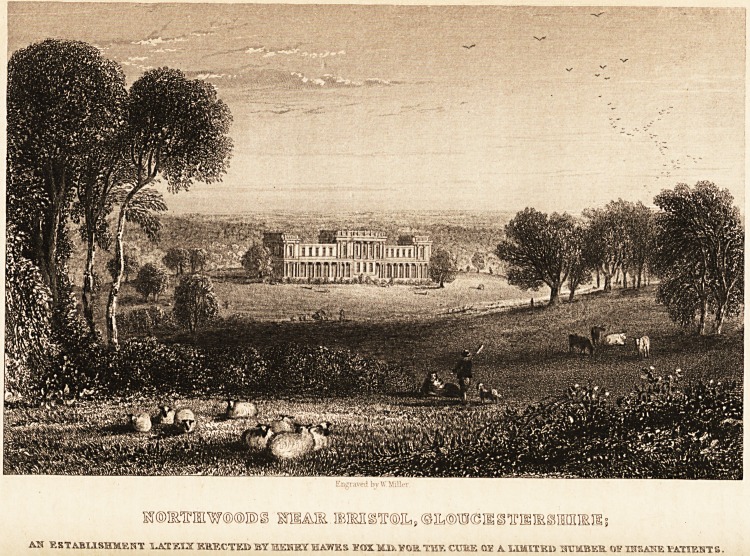# Extra Limites

**Published:** 1836-04-01

**Authors:** 


### EXTRA-LIMITES.

### NORTHWOODS LUNATIC ASYLUM.

We are requested to insert the following letter, containing a prospectus of a new establishment for the insane, lately erected by Dr. Fox, at Northwoods near Bristol.

Berkeley Square, *Bristol.*

I beg leave to submit to your consideration the following Prospectus, together with a sketch of a residence newly erected for the reception of a limited number of Insane Patients, and to be restricted to the more respectable classes of society. The building is finely situated upon an eminence, about seven miles from Bristol on the old road to Gloucester, has a south-eastern aspect, and combines great cheerfulness with perfect retirement. Considerable expense has been incurred in the architectural design, as well as in the internal decoration and arrangement, in order to remove from the minds of the Inmates the unpleasant impressions usually connected with such establishments.

In the *construction*, it has been a most important consideration to provide for perfect security from fire, by substituting iron joists and composition floors instead of wood. The fatal experience of at least two county asylums has rendered this precaution almost imperative.

The Panopticon principle has been adopted to the fullest extent compatible with comfort and security. The whole front of the building, which commands a beautiful and extensive prospect, is entirely devoted to the patients; and the superintendent's room is so situated as to allow the most constant and vigilant control to be exercised over the proceedings of the patients and their attendants, as would evidently appear upon a personal inspection. The most entire safety for those who may be resolutely bent on personal injury to themselves, forms a prominent feature in the internal arrangements; and such as reflect on the many valuable lives which have been sacrificed to the want of timely precaution, will know how to appreciate preventive measures.

The apartments of the patients are under the same roof, and in immediate connexion with the family, producing the desirable impression of a domestic circle. Particular attention is paid to the accommodation of invalids, whose delicacy of health requires extreme care and quietude, by means of cottages suitably provided for their reception. Almost adjoining the cottages, and at the distance of a quarter of a mile from the establishment, is a spacious, modern, family house, appropriated to the purpose of completing the recovery of invalids, whose convalescence is so far advanced as to render unnecessary the restrictions demanded by actually existing disease, but whose amendment is not sufficiently confirmed to justify a return to their families, without danger of relapse. The two buildings combine the greatest advantages for the permanent restoration of health, with the least possible pain to the feelings; since, as soon as the natural susceptibility to painful impressions returns, the invalid is removed from their contemplation.

Spacious and enclosed airing grounds and extensive colonnades attached to the building, afford ample opportunity for air and exercise; and with very few exceptions, arising from their peculiar state, the patients are permitted to range over the whole extent of the plantations, pleasure-grounds, and farm, accompanied by a careful attendant;—an advantage seldom attainable in a private dwelling.

The property is one mile in extent, and consists of two hundred acres of fertile land, without a public thoroughfare; affording great facilities for walking, riding, or carriage exercise, and the amusements of agriculture and gardening, without liability to personal exposure. Horses, with a close and an open carriage, are kept for the accommodation of patients, who are from station and habits accustomed to such resources.

The *treatment* of the patients is founded on a system of uniform kindness and tenderness, according to the principle so successfully pursued by that distinguished French physician, M. Esquirol. The limits of a Prospectus admit only a general description of the method of cure, of which the prominent features are the following:—*separation* of the patients from their immediate relatives and friends, whose kind intentions become too commonly sources of aggravation;—the *association* with invalids under similar circumstances of removal from their ordinary habits and connexions, which experience has proved to be a most important instrument of cure;—*constant occupation*, both agreeably and beneficially adapted to the state of the mind and body; together with such medical and moral treatment as may be most appropriate in each case. Seclusion in the chamber is the only restraint permitted, and this invariably requires the sanction of the superintendent. The most perfect division of the sexes, and the separation of those invalids whose state renders them unfit for social intercourse, from others whose welfare and comfort would be obstructed by indiscriminate association, have been objects of the most careful attention.

It is necessary to witness, in order duly to appreciate, the cheerfulness and complacency which, under such circumstances, generally characterise the social intercourse of the inmates.

The Establishment is under my own particular care and inspection, and the immediate superintendence of a widow lady, whose active benevolence, experience and judgment, peculiarly qualify her for the important undertaking.

The service of the Established Church is performed every Sunday, and those who are in a fit state, are permitted to join the family prayers every morning.

I may truly observe, that in the course of long experience, I have never known a person, however trustworthy, to whom an insane patient might safely be confided, without the control of a watchful superintendent to decide upon the necessity of restraint, and to regulate its degrees; indeed the power of constraint should never be entrusted to the personal attendant who has the immediate care of an insane patient. The convenience and interest of such persons are so constantly at variance with the welfare of the invalid; and it is so natural for them to persuade themselves of the expediency of measures, which appear to combine greater security to the patient with less trouble to themselves, that they are very inadequate judges, *however experienced*, of the course proper to be adopted. I am of opinion, that no case of insanity should be considered as incurable, without distinct evidence of mental imbecility: and that, as all disorders of the mind are more or less dependent upon bodily disease, beyond the immediate influence of medicine, most important benefits result from the power of uniform habits of exercise, diet, diversion and occupation, properly regulated, over the removal of such causes of complaint.

I am further anxious to impress on your especial notice, that the object of this establishment is the CURE of insanity, by means attainable only in such an institution; inasmuch as one of the most important and successful remedies depends upon the intimate association of persons brought together by a similarity of circumstances, calculated to beget mutual sympathy, regard, and confidence.

In conclusion, I beg to direct your particular attention to the acknowledged fact, that recoveries in a far greater proportion occur in well-regulated asylums for the insane, than under any other circumstances; that the building is not constructed to contain more than thirty patients; and therefore, independently of its peculiar arrangement for the purpose of constant superintendence, allows of a degree of particular watchfulness, which cannot exist in a very extensive establishment; and that I have been induced to engage in the present undertaking from a persuasion, as far as my knowledge and inquiries extend, that there is no establishment of a suitable description, where the class and number of the invalids are restricted; and that there has been long existing a demand for such an institution, to meet the necessities of the higher classes of society.

It may be satisfactory to mention, for the information of those to whom I am unknown, that I am a graduate of St. John's College, Cambridge; late President of the Royal Medical Society of Edinburgh; fifteen years Physician to the Bristol Infirmary; and more than twenty years Physician to St. Peter's Hospital, to which is attached the Lunatic Asylum for the city and neighbourhood of Bristol: from which last two offices I have not long since retired. Additional claim to confidence might be advanced, from the circumstance of my being the eldest son of the late Dr. Fox, of Brislington, the proprietor of an extensive Asylum for Lunatics, which has afforded me an opportunity of being intimately acquainted with the management of the insane from my earliest recollection; having dwelt under the same roof, and occasionally engaged in their care and direction.

I remain, Sir, your obedient Servant,

HENRY HAWES FOX.

*Address*—HENRY HAWES FOX, M.D.
Northwoods, near Winterbourne, Bristol.

---

*The following explanation and illustration of the effects of associating the Insane may not be uninteresting.*

That the greatest benefit accrues to the insane from their associating together, is a fact well established by experience, but one that speculative reasoning could hardly have anticipated. Once, however, clearly ascertained, it may admit of explanation, and, from the unfortunate prevalence of the malady, the inquiry is most important.

In partial insanity, which is the usual form of mental derangement in the chronic stage, the judgment is mostly unimpaired on subjects not connected with the prevailing illusion; and the feelings at the same time, instead of becoming obtuse, are more frequently exalted or rendered highly acute. From this it follows, where numbers are associated together, that each individual soon becomes aware of the errors of others, however slow to suspect his own. This discovery, which among the sane is too apt to call forth supercilious contempt, seems to awaken in a community of the insane only feelings of indulgence and commiseration. In many cases, the delicacy observed towards each other is such as will only be credited by those who have witnessed it. Now, while this kindness tends, on the one hand, to soothe the irritation of the invalid, the continual observance of each other's illusions has a tendency, on the other, to awaken in the mind of each a doubt regarding the soundness of his own views; for, notwithstanding the delicacy shewn to himself, he cannot fail to perceive that his own notions, on certain points, are esteemed erroneous by those around him. This doubt once awakened, naturally leads to self-examination, and this, if preceded by a restoration of the bodily organs to a healthful state of feeling and action, often terminates in conviction, and proves the first step to convalescence.

To effect this return to a healthful state of feeling requires however the combined influence of physical and moral treatment; the physical calling for knowledge and experience in the physician, and the moral for uninterrupted kindness and discretion in the attendants under his control. But this is far from all;—the moral treatment demands, first, entire seclusion from relatives and friends, thus breaking through former trains of thought and feeling; then perfect acquaintance in the physician with the character, temper, and disposition of each individual, which can only be gained by close attention, with the opportunities which a private asylum affords; to this must be added constant occupation or amusement of the patient to prevent his mind from dwelling on its own morbid impressions; and, those who have been accustomed to move in the higher sphere of life, require those comforts and conveniences which habit has rendered indispensable.

A striking illustration of the good effects of the association of the insane occurred in the course of this year by an invalid being placed under my care, who imagined, that the whole national debt was imposed upon him to pay, and in default of payment, that he was to be blown up with gunpowder! During the Summer, a gentleman from Devonshire, extensively engaged in commerce, was likewise committed to my charge, who had been insane during the preceding nine months, in consequence of his circumstances being somewhat impaired by the failure of a neighbouring bank. He imagined that he was involved to the extent of millions, and that he was irretrievably ruined. He was associated on the morning following his arrival with the former invalid, to whom he very fully detailed the history of his misery. He was heard with great attention and sympathy, and when he had concluded his narrative, the former invalid simply replied, "Pugh! pugh! pugh!" with a corresponding motion of the hand, "I owe trillions!" These persons continued to be associated during the three succeeding days, when the last invalid addressed his companion by remarking upon the folly of his notion with respect to the national debt, and conjuring him to forsake the idea; observing, that "I thought as you do three days ago, but I see it is all a fallacy, that there is no foundation for it, and that I am no more embarrassed than you are." From that moment, the gentleman from Devonshire relinquished his morbid hallucination, became well, and returned in a few weeks perfectly restored in health, both of body and mind—the former being very imperfect upon his arrival. It appeared that the last invalid was so forcibly impressed with the folly of the idea entertained by his companion, that it roused him to a more complete examination of his own mind, and produced the happy result of his almost immediate recovery.

## Figures and Tables

**Figure f1:**